# Monitoring dynamics of biocrust rehabilitation in acid-saturated desert soils

**DOI:** 10.1007/s10661-024-12865-y

**Published:** 2024-07-09

**Authors:** T. Kerem, A. Nejidat, E. Zaady

**Affiliations:** 1 Department of Natural Resources, Agricultural Research Organization, Institute of Plant Sciences, Gilat Research Center, Mobile Post Negev, 8531100 Gilat, Israel; 2 Department of Environmental Hydrology and Microbiology, Zuckerberg Institute for Water Research, The Jacob Blaustein Institutes for Desert Research, Ben-Gurion University of the Negev, Sede Boqer Campus, 8499000 Midreshet Ben-Gurion, Israel; 3 Kaye Academic College, 8414201 Beer Sheva, Israel

**Keywords:** Soil pollution, Biological soil crust, Rehabilitation, Contamination, Acid water, Industrial by-products

## Abstract

The study explores the aftermath of a wastewater reservoir failure in a phosphate fertilizer industry, resulting in the release of acidic water containing phosphorus and sulfate compounds into the Ashalim stream’s Nature Reserve in the Judean desert, which affected the soil surface biological crusts (biocrusts) layer. The study aims to examine contamination effects on biocrusts over 3 years at two research sites along the stream, compare effects between contaminated sites, assess rehabilitation treatments, and examine their impact on soil characteristics. Hypotheses suggest significant damage to biocrusts due to acidic water flow, requiring human intervention for accelerated restoration. The results indicate adverse effects on biocrust properties, risking its key role in the desert ecosystem. The biocrust layer covering the stream’s ground surface suffered significant physical, chemical, and biological damage due to exposure to industrial process effluents. However, soil enrichment treatments, including biocrust components and organic material, show promising effects on biocrust recovery.

**Supplementary information:**

The online version contains supplementary material available at 10.1007/s10661-024-12865-y.

## Introduction

The Ashalim stream basin, with its nature reserve, is an essential ecological corridor connecting the Judean and the Negev Deserts (Fig. S9). It is a strip of land allowing biotic factors (wildlife) to move between the two regions. It is crucial in conserving these two ecosystems’ biodiversity and appropriate functioning (Belote et al., 2016; Haddad et al., 2003). Blockading this corridor prevents many species from accessing their life support systems (e.g., water and food resources, reproduction, and shelters) (Ward et al., 2020).

**Fig. 1:**
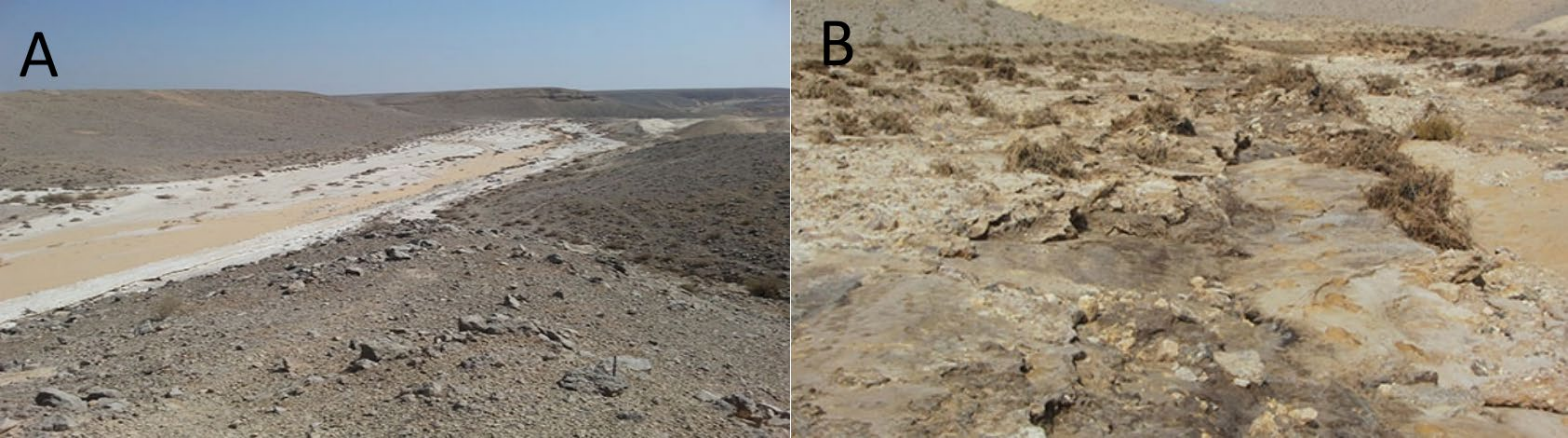
An overview of Ashalim Nature Reserve after flooding in the sandy part of the stream with the wastewater (**A**) and a close view of the destructive impact on the mainstream (**B**) as observed on July 3, 2017, a few days after the event

On June 30, 2017, approximately 150,000 m^3^ of phosphate fertilizer’s industrial by-product process wastewater flowed into the Ashalim stream’s Nature Reserve (Even-Danan et al., 2014; Tzoar, 2018) (Fig. 1). The gush occurred due to a wall failure of one of the wastewater reservoirs of a nearby phosphate fertilizers industrial plant. A high volume of acidic water contaminated with phosphorus and sulfate compounds and other chemical elements (Table S1) flew into the mainstream and its margins (Fig. 1). This tragic event prompted a monitoring study to evaluate its impact on the soil surface while considering strategies to restore the ecological corridor that was functionally disconnected following the event.

Due to the flooded wastewater’s high acidity (pH 2.5–3), we anticipated severe damage to the soil surface biota. In this fragile hyper-arid ecosystem, the soil surface layer is known as bio-crusts (biocrusts, biological soil crusts). Biocrusts cover large portions of the soil surface worldwide in hot and cold dry regions (Belnap, 2003; Bowker et al., 2018; Weber et al., 2016). In these ecosystems, biocrust-dwelling microorganisms function as ecosystem engineers and provide critical services (Szyja et al., 2019) and essential functions, including primary production and, together with the absorption of aeolian dust, enrich the soil with growth-needed nutrients (Zaady & Offer, 2010). In addition to soil enrichment with organic matter and fixed atmospheric nitrogen, biocrust formations prevent soil surface erosion by overland run-off (during rainfall events) and wind (Barger et al., 2016; Eldridge et al., 2002; Belnap, 2003; Belnap, 2006; Chamizo et al., 2016; Faist et al., 2017). Biocrusts are susceptible to natural and anthropogenic disturbances, such as mechanical trampling, crumbling, powerful rinsing, biological sensitivity to pollutants, and sharp changes in soil conditions such as pH, nutrient content, and soil water regime (Barger et al., 2006; Belnap, 2006; Weber et al., 2016; Zaady et al., 2016a; Zhao et al., 2016). The vulnerability of biocrusts to anthropogenic disturbances may persist for many years, leading to soil surface degradation (Belnap & Eldridge, 2003). Rehabilitation processes may preserve and improve the functional recovery of degraded biocrusts (Belnap, 2003). Furthermore, the ecotoxicological effects of the contaminant may have disturb the dynamic equilibrium of the microorganisms in the soil surface and thus jeopardizing the ecosystem functioning and rehabilitation (Glenn & Dilworth, 1991; Rousk et al., 2009; Raaijmakers et al., 2009; Li et al., 2017).

The ecotoxicological effects of acid mine drainage on surface water, stream sediments, and macrophytes are well known (Alvarengaet al., 2021; Lucas et al., 2021). However, the effects of acid mine drainage pollution on biofilm continuity, function, and restoration in hyper-arid climates are unclear. The contamination event in the Nature Reserve Ashalim stream provides a unique opportunity to examine these processes under natural conditions and with the support of human intervention.

We hypothesized that (1) the biocrusts covering the channel’s soil surface experienced significant damage, both mechanically due to flow in an abnormal volume and biologically and chemically due to exposure to the industrial process wastewater; (2) due to the conditions created after the event, the biocrusts’ restoration in the hyper-arid area may take decades; (3) human intervention can accelerate the rate of restoration of biocrusts; (4) reestablishing of the biocrust constitutes is a primary stage in restoring the ecosystem in the impacted area.

Accordingly, the study’s objectives were to examine the effects of contamination events in the Ashalim stream on biocrust structure and functioning along the sandy upstream and the alluvial lower sections while monitoring the natural recovery and the impact of artificial rehabilitation actions. We aimed (1) to examine the effects of the contamination on the biocrusts in the local ecosystem; (2) to compare two different research sites (with different soil properties and distances from the breakthrough point of the reservoir) along the stream and examine whether the intensity of the impact is the same between the two; (3) to compare the efficiencies of different primary rehabilitation strategies (such as to that of natural rehabilitation processes); and (4) to examine the impact of restoration treatments on additional traits in the ecosystem, as chemical and physical soil characteristics and biodiversity.

## Methods and materials

### Study site

The Ashalim stream is a medium-sized channel and flows from the Judean desert’s southern part (~ + 390 AMSL) to the Dead Sea Valley (~ − 400 BMSL) in a winding line of about 20 km. The watershed area of the river’s drainage basin is about 80 km^2^. Most of the annual rainfall is during the winter season (November to April). The upstream average rainfall is about 70 mm per year, and at the lower part, about 40 mm per year (Online Resource Fig. S10, Israeli Meteorological Service. (2024)).

We established two sites in the Ashalim stream to monitor natural recovery processes and implement rehabilitation treatments. (a) The first site is in the stream’s high catchment basin, characterized by sandy soil (Torriorthents according to the USDA system and Sandy-regosols according to the local Israeli classification system (Dan et al., 1975). (b) The second is an alluvial site at the bottom of the stream (Xerochrepts according to the USDA system and Alluvial-arid-brown-soils according to the local Israeli classification system (Dan et al., 1975). For the sandy part, we selected a damaged section (designated ASH1) in close vicinity of the damaged reservoir (31°04′ 29.5″ N; 35°13′ 30.2″ E). The site is located at about + 370 a.m.s.l. As a control, a sandy section of the Gmalim stream (GML) (31°05′ 30.7″ N; 35°13′ 50.2″ E), about 1.5 km north of ASH1, was chosen. The GML stream was not exposed to the contamination event, so this site is suitable as a control and has mature undisturbed biocrusts typical to this climatic condition. For the alluvial part of the Ashalim stream, a contaminated site (ASH2) was chosen (31°03′ 53.3″ N; 35°19′ 55.1″ E). The site is located at about − 260 b.m.s.l. The channel is relatively narrow in this part of the stream, typical of this geographical area. As a control, the ASH3 site was chosen (uncontaminated section) and located on the alluvial shelf, about 20 m above the stream channel and about 500 m east of the ASH2 site (31°03′ 35.4″ N; 35°20′ 31.9″ E). The control plot is identical to the contaminated part, is richer in clay soil, and has mature biocrusts (Online Resource Fig. S11).

The study was conducted for 2 years. Soil surface samples (zero time) were collected at a depth of 1 cm from the plots before applying the treatments. The samples were then analyzed to determine the chemical profile (Table S2), the soil’s characterization, and its physical and biological properties. Field testing and sampling were set for the spring season, and the soil surface samples were transferred in ice bags for laboratory analyses.

### Treatments

Five plots were marked on each site (ASH1, GML, ASH2 and ASH3). Each plot was divided into five sections of 1 m^2^ and subjected to five different treatments (Online Resource Fig. S12). The first two treatments monitored the existing condition in the field. They examined the natural restoration over two seasons, and up to 3 years after the event. In treatment (1), soil surface remained uninterrupted (N), while in (2), the soil surface crumbled (B). In treatment (3), the surface was inoculated with the filamentous cyanobacteria *Microcoleus vaginatous* (I), isolated from the uncontaminated control areas. This cyanobacterium is commonly the first to appear after biocrust disturbance and plays a crucial role in the succession of biocrusts (Belnap, 2001). Growing large masses under laboratory conditions allows for application in large quantities to the soil surface. The *M. vaginatous* was grown in a bg-11 medium (http://microbiology.ucdavis.edu/meeks/BG11medium.html). The cyanobacterial fragments were harvested by filtration, dried in the oven at 45 °C for 48 h, ground to a powder, and kept at 4 °C until application. We added 10 g of dry cyano-powder to the appropriate treatment (1 m^2^). In treatment (4), we applied two methods of re-inoculating the soil surface with biocrust components (C) collected from the parallel control site. In this treatment, biocrust components collected in the GML stream for the upper site and at ASH3 for the lower site were dried, sieved to keep large particles away, using a 2-mm sieve, and then applied at 10 gr/m^2^ to the experimental plots (Fig. S12). In treatment (5), a mixture ground straw of wheat and legume was added (O). This rehabilitation treatment was tested in various parts of the world with high success rates (Wang et al., 2008; Belnap, 2003). This treatment aimed to examine the effect of adding organic matter to the topsoil layer. Organic matter is an essential component in soil formation and affects its physical and biological properties (Lai et al., 2013; Ros et al., 2003). A mixture of wheat and legume straws (*Triticum vulgare* and *Pisum sativum* 1:1 ratio) was ground (> 1000 µm), and 1 kg was applied by mixing with the upper soil layer to a depth of 10 cm. All plots were sprayed in 1 l of double distilled water (equal to 1 mm of rainfall) to give an equal starting point and stabilize the additions given to the soil surface.

Chronically, the contaminated event occurred in July 2017. The reference sampling was performed in 2018 (Online Resource Table S2), and the rehabilitation results were documented during 2019 and 2020. The reference field tests were conducted about 2 weeks after the last rainfall event of the rainy season (April 2018). From each of the rehabilitation treatments treatment, three composite samples of biocrusts were collected randomly (Zaady & Offer 2010) on two dates: January 2019 and the other in April 2020. The Cornell Soil Health Test protocols were adopted for analyzing 14 physical, biological, and chemical soil properties (Gugino et al., 2009). The physical properties included soil texture (fractions of clay, silt, and sand), surface hardness (SH), and hydraulic conductivity (HC). The biological properties included soil organic matter (SOM), potential organic carbon (POC), polysaccharides, and chlorophyll (*a*). The chemical properties included soil pH, electrical conductivity (EC), Olsen phosphorus (P), extractable potassium (K), fluoride (F), soil ammonium (N-NH_4_^+^), and soil nitrite (N-NO_2_^−^).

### Chemical measurements

Fluoride concentration was determined by extracting soil moisture in a ratio of 1:10 from soil collected in December 2018 in the Ashalim stream (the Dead Sea Research Institute, Israel). Potassium was determined by calcium chloride (Simonis et al., 1996). The soil’s phosphorus availability level was tested using the Olsen method at a wavelength of 880 nm (Olsen & Summers, 1982; Kuo, 1996).

Biocrust organic matter (OM) content was determined by combustion at 450 °C, after washing in 0.1 N HCl (Ben-Dor & Banin, 1989). Potential organic carbon (POC) was measured according to Tirol-Padre et al. (2004) at a wavelength of 550 nm. The amount of active carbon (dissolved) in the samples was calculated according to the following equation:

$$POC(\text{mg }{\text{g}}^{-1})=\frac{\left(\text{A Sample}\right)*\left(50/2\right)*25*9}{1000 \left(\text{mL }{\text{L}}^{-1}\right)*\text{wt of sample }(\text{g})}$$

where *A sample* is KMnO_4_ solution concentrations in the sample calculated by the value of the calibration curve. 50/2 is the dilution factor; 25 is the quantity in ml of KMnO_4_ solution added to the soil sample; 9 is the amount of carbon dioxide on each mol KMnO_4_ (see Tirol-Padre et al., 2004).

For determining the inorganic nitrogen concentrations, soil samples were extracted in 1 M KCl (2.5 g in 10 ml, for 60 min) and filtered through 0.45-µm filters. Soil ammonium-nitrogen (N-NH_4_) was tested by calorimetric analysis with Nessler reagent, at a wavelength of 420 nm (APHA, 1989). Nitrite-nitrogen (N-NO_2_^−^) was measured by colorimetric analysis with Diazotizing reagent at a wavelength of 543 nm (APHA, 2005), using a Tecan Infinite® 200 PRO spectrophotometer (M¨annedorf, Switzerland). Soil nitrate-nitrogen test (N-NO_3_^−^) was measured by the second derivative method (Ferree & Shannon, 2001).

The concentration of ammonium-nitrogen and nitrite-nitrogen was calculated according to the following equation:

$$\text{N}-\left({\text{NO}}_{2}\right)/\text{N}-\left({\text{NH}}_{4}\right)=\left(\text{A sample}\right)\times 4$$

where *A sample* represents the nitrogen concentration in the sample and 4 is the dilution factor.

### Physical measurements

Soil electrical conductivity (EC) and pH were measured by shaking 1 h of 10 g of soil in water (1:1 ratio) and then centrifuging for 10 min at 4500 RPM, and the dilution was filtered (Whatman 42). The EC values (dS/m) using a total dissolved salts (TDS)/conductivity meter (Cole-Parmer, Vernon Hills, IL, USA). A penetrometer’s breaking strength of the soil surface, measuring the force required to penetrate the biocrusts (Pocket Penetrometer Eijkelkamp company). The device’s measuring units are kg/cm^2^, and the reading range is 0–4.5, performing ten readings for each study treatment.

The infiltration rate varies depending on the soil’s physical properties, mechanical composition, organic matter content, and saturation state. The water’s infiltration rate was determined by the soil’s hydraulic conductivity and indicated the soil’s ability to conduct fluid in a unit of time. A mini-disk infiltrometer device (Decagon Company) was used. Based on the Van Genhuchten equation, converted water permeability data were to unsaturated soil’s hydraulic conductivity values (Zhang, 1997; Naik et al., 2019). A shear gauge measured soil surface shear force. The surface’s stability affects the soil surface’s shear force (Pijket Shear vane tester, Eijkelkamp Company). It is a significant factor in preventing soil erosion during the rainy season. The test was conducted at the research sites, and ten readings were performed for each study treatment. The measuring units of the device are kg/cm^2^, and the reading range is 0–10.

A volumetric soil sample filtered through a sieve (2 mm) to separate small stones was dispersed in a Graham salt solution (pyro-phosphate) at a concentration of 5% for 12 h. The dispersed soils were transferred to the bowl of the Mastersizer, which performs a volumetric mechanical particle test (A 3000 Mastersizer device manufactured by Malvern). The masterizer performs a self-calibration between each test. The instrument tested each soil sample four times, and the average results were obtained (Eshel et al., 2005).

### Bio-physiological measurements

Biocrusts’ presence and development were determined by using three different bio-physiological methods; chlorophyll *a*, and polysaccharide content (Zaady et al., 2016b). The chlorophyll was extracted with ethanol, and the extracts were evaluated with a UV-VIS mini-1240 spectrophotometer (Shimadzu, Colombia, MD, USA: Castle et al., 2011). The absorbance wavelength for measuring chlorophyll *a* was calculated according to Lichtenthaler and Wellburn (1983), as follows:

$$chll a\left(\frac{\mu \text{g}}{\text{ml}}\right)=13.7\times \left(A \left(665 \text{nm}\right)-5.76\times \left(A \left(649 \text{nm}\right)\right.\right)$$

where *A* represents the wavelength used.

Polysaccharides were assessed using anthrone reagent and sulfuric acid, a modification of a method developed by Chamizo et al. (2018) and Fedrico et al. (2012).

The percentage of coverage test is an observational measure of the biocrusts’ presence in the study plots. The test was performed using a metal mesh (Online Resource Fig. S13), dividing each square meter into 25 equal-sized squares (400 cm^2^). In each 1 m^2^, an assessment was made of the biocrust cover as well as the amount of scale, sand, and vegetation.

### Statistical analysis

A Oneway Anova (one-way analysis of variance) test was performed to compare the chemical, physical, and biological parameters within the research sites and the different treatments. Each repetition contained three samples that merged. Therefore, three returns for analysis from each treatment contained an average of nine collections. This analysis allowed us to obtain information on the effectiveness of treatment at each site individually, with N (natural) treatment being an internal control treatment.

In addition, the chemical, physical and biological parameters between the sites were measured, in the different years, by the ANCOVA test, where the experiment design consists of two or more factors (full factorial), and the effect of the block and the interaction (block * site) is random. The measurement values obtained in the first year (zero year–2018) were included in the covariant model. This test allowed us to get information about the differences between the different sites and between the years. Before performing this statistical test, the Levene test was performed to test for equality of variance.

An ANCOVA test was conducted, in the same design, using a separation according to the control or the contaminated site. Here, the measurement values obtained in the first year (2018) were included in the covariant model. This test allowed us to get information on the treatments’ effectiveness according to the source of the site and to find differences in the behavior of the treatments at the control sites versus the contaminated sites. All statistical analyses were performed in the statistical software JMP 14 (SAS Institute Inc., Cary, NC, USA).

## Results and discussion

### The effect of treatments between sites

Monitoring natural soil rehabilitation while actively improving soil surface may suggest an integrated framework for soil restoration. Our results reveal that the catastrophic environmental event in Ashalim Nature Reserve in the Judean desert severely damaged the biocrust layer due to the by-product acidic water flood. It was important to use indicators pointing out the positive biocrusted soil surface development process to analyze the damage and the possibility of recovery. We used physical, chemical, and biological parameters and qualitative biological indices (Gugino et al., 2009; Zaady et al., 2013, 2016b, 2022).

Weber et al. (2016) suggested that soil conditions and disturbance severity influence the natural recovery of biocrusts. Furthermore, biocrusts are sluggish to recover, and the process is highly dynamic, especially in hyper-arid ecosystems. The successional development can be varied by geodiversity, climatic conditions, and the intensity of the disturbances (Salminen et al., 2023; Zaady et al., 2022).

Several methods were suggested in the scientific literature for obtaining biomass of biocrust for reclamation of disturbed areas to encourage the recovery of the biocrust components to construct the soil biological crusts. Most procedures are based on the collecting natural biocrust components from the habitat area and their dispersal in the disturbed areas for rehabilitation, which can hasten the recovery of their structure and function (Maestre et al., 2006; Chiquoine et al., 2016; Condon & Pyke, 2016; Zhao et al., 2016). This process is much more complex in drylands where water scarcity and the land are more fragile and vulnerable to degradation (Orr et al., 2017; UNCCD, 2017; FAO, 2022, 2023; Levi et al., 2021). We included this suggestion in the current study and, in addition, considering the possibility that in the case of additional assisting factors located in the rhizosphere of biocrusts at the GML and ASH3 sites, we collected biocrust aggregates (from a depth of up to 1 cm), which we used for treatment [C].

There are several considerations in choosing a particular species from the biocrust community, which will constitute a key species in the restoration operations. Belnap and Büdel (2016) suggested that cyanobacteria are the first colonizers of terrestrial ecosystems as inoculants to encourage biocrust development to restore degraded arid areas. In hyper-arid conditions, like in the southern Judean Desert, where rainfall is rare (Online Resource Fig. S10), only the primary stage of the biocrust succession can be observed. Another consideration is the degree of adaptation of the chosen species to the target environment, with species already existing in this environment having priority because of their local adaptation level. Therefore, we isolated the dominant filamentous cyanobacteria *Microcoleus vaginatus* from the GML and ASH2 sites and used it for inoculation for restoration [treatment I] (Xiao et al., 2023).

The reserve is in a hyper-arid region that does not contribute to fast rehabilitation since water is an essential component for biocrust development and encourages natural restoration (Wang et al., 2008). The soil was not moist enough during the biocrust rehabilitation stages, and slow rehabilitation is verified by the results obtained in this study.

### Chemical parameters

As mentioned above, biocrust samples were collected on three dates. The first sampling was conducted in December 2018 where the biocrust was tested before setting the rehabilitation treatments (served as a reference point), followed by sampling the treatments in 2019 and 2020.

**Fig. 2:**
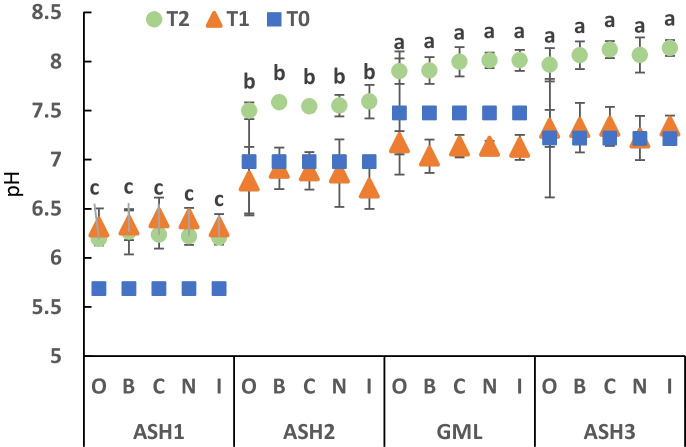
Comparison of the soil pH of the soil between the treatments* in the four research sites. The columns show the mean ± standard error (*n* = 3). The pH of T1 and T2 represents the sampling of 2019 and 2020, respectively. T0 is the reference sampling in December 2018. The letters represent a significant difference (*p* < 0.0001) between the first ANCOVA test sites. *Restoration by application of organic matter in the plot [O], local disruption of the biocrust layer [B], application of biocrust components collected in the control area [C], natural rehabilitation [N], and rehabilitation by application of the cyanobacterial biocrust components isolated and grown in the laboratory [I]

In ASH1, the pH in 2018, 1 year after the event, was 5.7 and increased to about 6.2–6.3 in 2019 and 2020. The pH in ASH2 was 6.8–7 in 2018 and 2019 and increased to 7.5 in 2020. The pH of GML (control upper stream) and ASH3 (uncontaminated site) was higher than the contaminated sites, at the range of 7.2 and 7.5 in 2018 and 2019, and increased to about 8.0 in 2020. No apparent differences were found between the treatments within each site (Fig. 2).

The ASH1 site showed significant differences between T0 (2018) compared to T1 and T2 (2019 and 2020, respectively). In ASH2 and ASH3, the pH values of T2 were higher than those of T0 and T1. The same for GML samples, pH values of T2 were higher than T0 and T1.

Considering the chemical parameters pH and EC tested, the change in the upper soil solution is evident in the affected sites. The influence of the acidity of the industrial process water weakened over time. Nevertheless, both sites, ASH1 and ASH2, are lower than their control sites, GML and ASH3 (respectively). Interestingly, the relatively small floods that occurred in the years following the event might have helped improve pH and EC. These results could be related to the fact that the soil surface washing processes and their strength are different between the two sites. The ASH1 site is located at the upper point of the Ashalim stream, while the ASH2 site is located at the lower point of the stream. The catchment basin of ASH1 is small (about 0.025 hectares), while that of ASH2 is more than 50 times larger since it drains more water along the stream from both sides, including secondary channels.

**Fig. 3:**
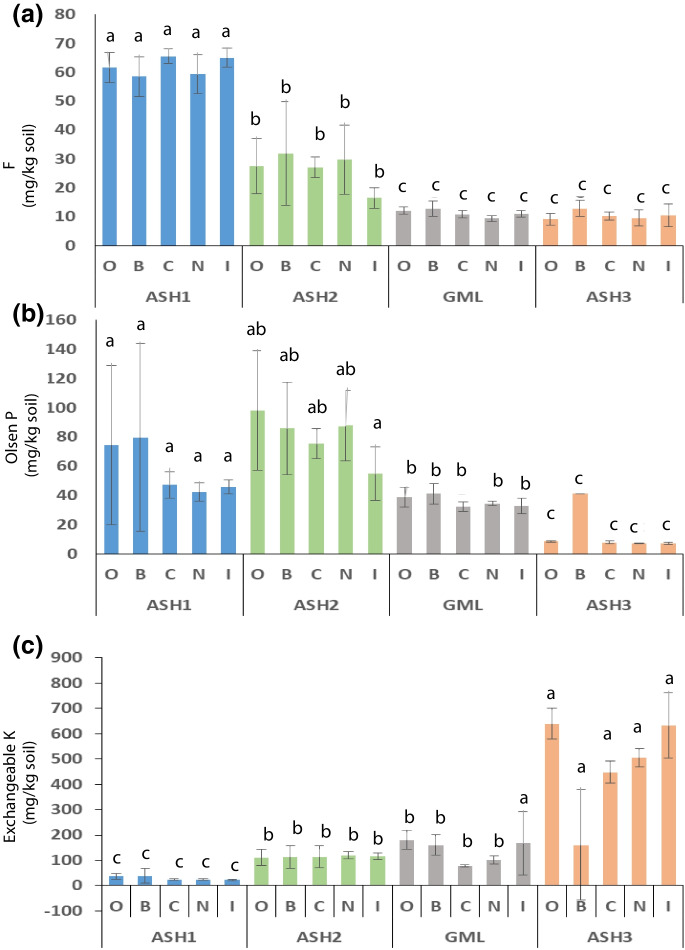
Comparison between the treatments* at each site in 2020; **a** the concentration of fluorine, **b** the concentration of phosphorus, and **c** the concentration of potassium. Different letters represent a significant difference. The columns and the error bars represent mean ± standard error (*n* = 3), indicating a specified confidence interval for each parameter between the sites (*p* < 0.0001). *Restoration by application of organic matter in the plot [O], local disruption of the biocrust layer [B], by application of biocrust components collected in the control area [C], natural rehabilitation [N], and rehabilitation by application of the biocrust components isolated and grown in the laboratory [I]

The elemental contents of fluoride, potassium, and phosphorus in the biocrusts samples were determined. Higher levels of fluoride (Fig. 3a) and phosphorus (Fig. 3b) were found in the contaminated ASH1 and ASH2 sites (Fig. 3, Table S1) (*p* < 0.0001). In December 2018, soil nutrient analysis showed elevated phosphate levels in the contaminated stream (ASH1, ASH2), with higher potassium (Fig. 3c) and sodium levels in the GML site (Online Resource Table S2). Narrow shelf samples in the lower control plot (ASH3) had significantly higher potassium levels compared to other samples (*p* < 0.0001) (Fig. 3). ASH1 had significantly lower values than ASH2 and GML sites. However, no differences were observed among the treatments within sites (Fig. 2).

**Fig. 4:**
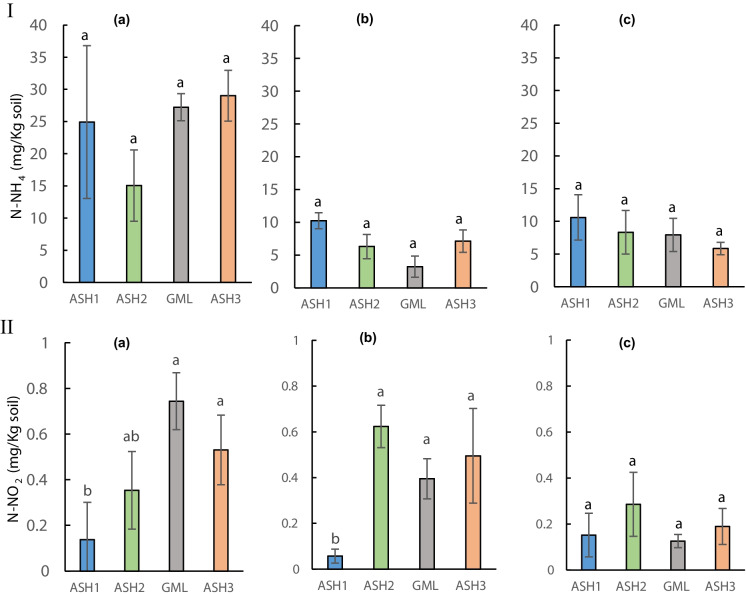
Comparison between the sites of soil-ammonium (**I**) and soil-nitrite (**II**), December 2018 (a), 2019 (b), and 2020 (c). The columns show the mean ± standard error. Different letters represent a significant difference

The differences in the N-NO_2_ and N-NH_4_ concentrations in the upper soil surface layer (< 1 cm, biocrusts development layer) might be related to the fact that while N-NO_2_ might move within the soil solution, while the N-NH_4_ is accumulated (Fig. 4). These results could be related to the fact that the soil surface washing processes and current strength are different between the two sites, giving an advantage to these processes in the ASH2 site compared to the ASH1 site. Nevertheless, the soil N-NH_4_ in the ASH1 and ASH2 continue to show a damage phase.

A preliminary examination of the results found no notable differences between the different treatments at each of the two sites tested ASH1 and ASH2. However, in some of the illustrations, differences were found in some parameters tested between the years. The tested N-NH_4_^+^ variable indicated an increase in concentration between 2019 and 2020 at two sites in the channel (Online Resource Fig. S14). This, compared to N-NO_2_^−^. It may be related to the possibility of leaching N-NO_2_^−^ in the soil solution compared to N-NH_4_^+^.

The ANCOVA test, which was divided according to the type of site, found no significant effect of the treatments within N-NH_4_^+^ at the four sites (Fig. 4I, Online Resource Fig. S14 panel Ia,b, Table S1). However, a significant change trend in N-NH_4_^+^ (*p* < 0.0001) was seen during the two sampling seasons within the treatments. While in N-NO_2_^−^, which was divided according to the type of site, no significant effect of the treatments at the four sites was found (Fig. 4II, Online Resource Fig. S14 panel II a,b). Nevertheless, a significant change in N-NO_2_^−^ (*p* < 0.0001) was seen during the two sampling seasons within the treatments at the control sites (Online Resource Fig. S14 panel II b).

**Fig. 5:**
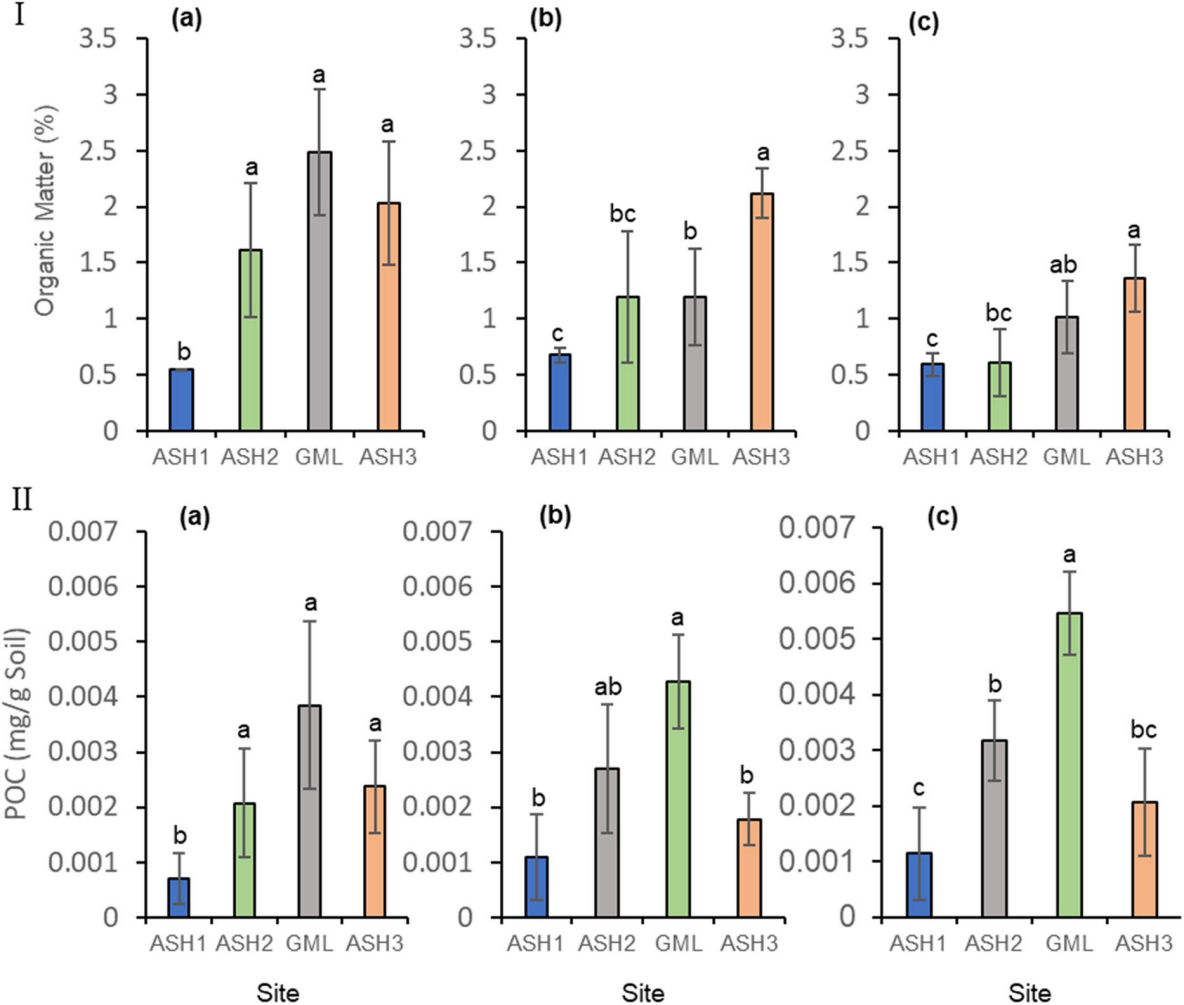
Comparison between sites regarding soil organic matter (**I**) and the potential organic carbon – POC (labile carbon) (**II**). December 2018 (a, reference sampling), 2019 (b), and 2020 (c). The columns show the mean ± standard error. Different letters represent a significant difference

It was suggested that during the biocrust successional development, soil nutrient and organic matter contents increase (Weber et al., 2016). Therefore, we added organic matter as our treatment [O]. In 2018, the organic matter percentage and the POC minor increase in ASH1 in comparison to high levels in GML site, whereas in ASH2 was not significant with ASH3 site. The years 2019 and 2020 showed similar results—significantly lower in ASH1 compared to GML site and ASH2 to ASH3 site (Fig. 5). Although lower values, a significant positive effect of adding the soil organic matter with treatment [O] was found at the ASH1 site (*p* < 0.0001) in comparison to treatment [N] (Online Resource Fig. S15 panel Ib), with similar results obtained in the GML site. No significant differences were obtained between the treatments in the other sites. However, we found a difference between the two seasons in the ASH3 site (Online Resource Fig. S15 panel I b). Considering the POC levels, in the ANCOVA test divided according to the type of site, a significant effect of treatment (O) was found at the ASH1 research site (*p* < 0.0001) in comparison to the other treatments in T2 (2020), except treatment [C] in T1 (2019) (Online Resource Fig. S15 panel II a). The other treatments did not show a significant effect (Online Resource Fig. S15 panel II a,b).

Larney and Angers (2012) examined how organic amendments affect soil properties (physical, chemical, biological). They showed that an extensive application of organic amendments can accelerate initial inactivation and lead to self-sustaining net primary productivity. We found that a significant positive effect of adding soil organic matter, [treatment O], was found at the ASH1 site compared to the [N] treatment (Online Resource Fig. S15). A similar result for adding the organic matter was indicated also on the values of the available POC. A positive result was not obtained at the other sites. This can be explained by the fact that site ASH1 is sandy, closest to the breached pond, and was vigorously washed by the acid water flood. The other three sites initially contained some organic matter. Site GML is a natural site with biocrusts and a sporadic distribution of annuals and perennials. Site ASH2 is far downstream from the starting point of the flood and is colonized by perennial shrubs that survived the event. Site ASH3 is rich in thick biocrusts that were not damaged by the flood (Fig. S12).

### Physical parameters

**Fig. 6:**
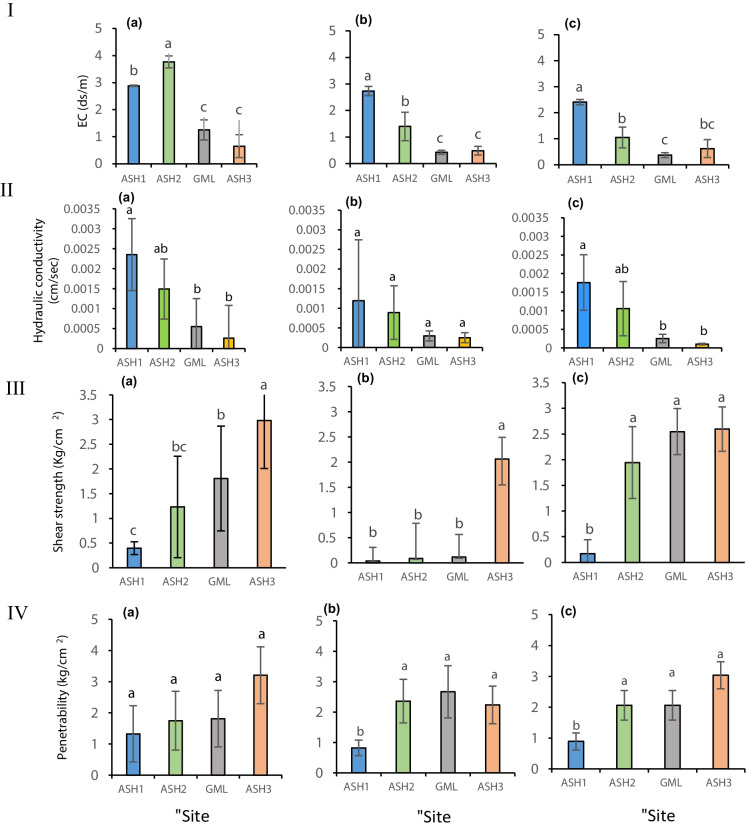
Comparison of the EC (**I**) (soil-water extraction in a 1:1 ratio), HC (**II**), penetrability strength (**III**), and the shear force (**IV**) of the soil surface in the four research sites. Results of December 2018 represent a reference sampling (a), 2019 (b), and 2020 (c). The columns show the mean ± standard error. Different letters represent a significant difference

Chamizo et al. (2016) and Belanp (2006) reported that biocrusts strongly influence dryland hydrological processes by modifying soil properties that affect soil water infiltration rate. These characteristics can be altered dramatically with soil surface anthropogenic disturbance (Alvarengaet al., 2021; Eldridge et al., 2002; Faist et al., 2017; Lucas et al., 2021; Zaady et al., 2016a). The by-product of acidic water flood in the Ashalim stream, which drastically affected biocrust coverage, significantly influenced HC and EC (Fig. 6I, II). The pattern found was similar to both parameters. The flood increased the EC in the soil surface by depositing salts such as phosphogypsum and others (Online Resource Table S1). The high values of HC indicate that the soil surface is more porous. The results showed persistent damage in ASH1 in the sandy part of the stream, while ASH2 in the alluvial part showed no difference from the control. Furthermore, it can indicate an improvement in the properties of the biocrust in ASH2 with time (Fig. 6II).

In addition to soil compositions (Online Resource Fig. S11), some other soil physical properties such as electrical conductivity (EC), hydraulic conductivity (HC), breaking strength (penetrability), and the shear force strength of the soil surface were examined. The EC values of ASH1 and AHS2 (the two contaminated sites) were significantly higher (*p* < 0.0001) than those in the control sites (GML and ASH3) in 2018 (a), 2019 (b), and 2020 (c) (Fig. 6). ASH1 results were significantly higher than GML ones in 2019 (b) and 2020 (c). Similar results were obtained between ASH2 and ASH3 in 2018 (a) and 2019 (b). However, no significant differences were measured between EC values of ASH2 and ASH3 in 2020 (c) (Fig. 6I). The ANCOVA test, which was divided according to the type of site, did not indicate any significant effect of the treatments on the soil’s EC (Online Resource Fig. S16).

The HC showed a similar pattern to EC. ASH1 is significantly higher (*p* < 0.0001) than that in the control sites (GML and ASH3) in 2018 and 2020 (Fig. 6Ia, Ic, respectively), while no differences were found in 2019. The ANCOVA test, which was divided according to the type of site, did not indicate any significant effect of the treatments on the soil’s HC (Online Resource Fig. S8). Furthermore, no statistical differences for HC were obtained between the treatments at all sites (figure not shown).

Fine soil particle (e.g., silt and clay) contents of encrusted soils increase with biocrust growth (Belnap, 2001; Belnap & Büdel, 2016; Zaady & Offer, 2010; Zaady et al., 2016a, 2016b). However, because of the flood intensity in the sandy ASH1 site, a high amount of moving sand and low fine particles accumulate (Online Resource Fig. S11). The damage manifests in the soil surface’s weakness and the nearly minimal development of the biocrust layer. The above effects can be seen in the results obtained in the physical properties, showing that the breaking strength and the shear strength of the soil crust in the ASH1 and ASH2 sites are still affected by the catastrophic event and dissimilar from their controls. The stability of the soil surface layer is still low in ASH1.

Considering the shear force strength of the soil surface, the ANCOVA test, divided according to the type of site, showed a significant effect between ASH3 and ASH2 in 2018 and 2019. Similar differences were obtained between the GML and ASH1 sites in 2018 and 2020 (*p* < 0.05) (Fig. 6III). A profound difference (*p* < 0.0001) was found between 2019 and 2020 in both ASH1 and the GML control site (Online Resource Fig. S17 panel I a,b). Furthermore, a significant change (*p* < 0.0001) was found between the treatments O and B in ASH1 in 2019 and 2020. Interestingly, a similar pattern was obtained in the ASH3 site. The penetrability strength of the soil surface showed no differences between the sites in 2019 and 2020 (Fig. 6II). However, the GML and ASH3 sites were higher than ASH1 and ASH3 sites (respectively) (Online Resource Fig. S17 panel II a,b). Similar to the pattern for the shear force, a significant change (*p* < 0.0001) was found between the treatments O and B in ASH1 in 2019 and 2020 (Online Resource Fig. S17 panel IIb).

### Bio-physiological parameters

**Fig. 7:**
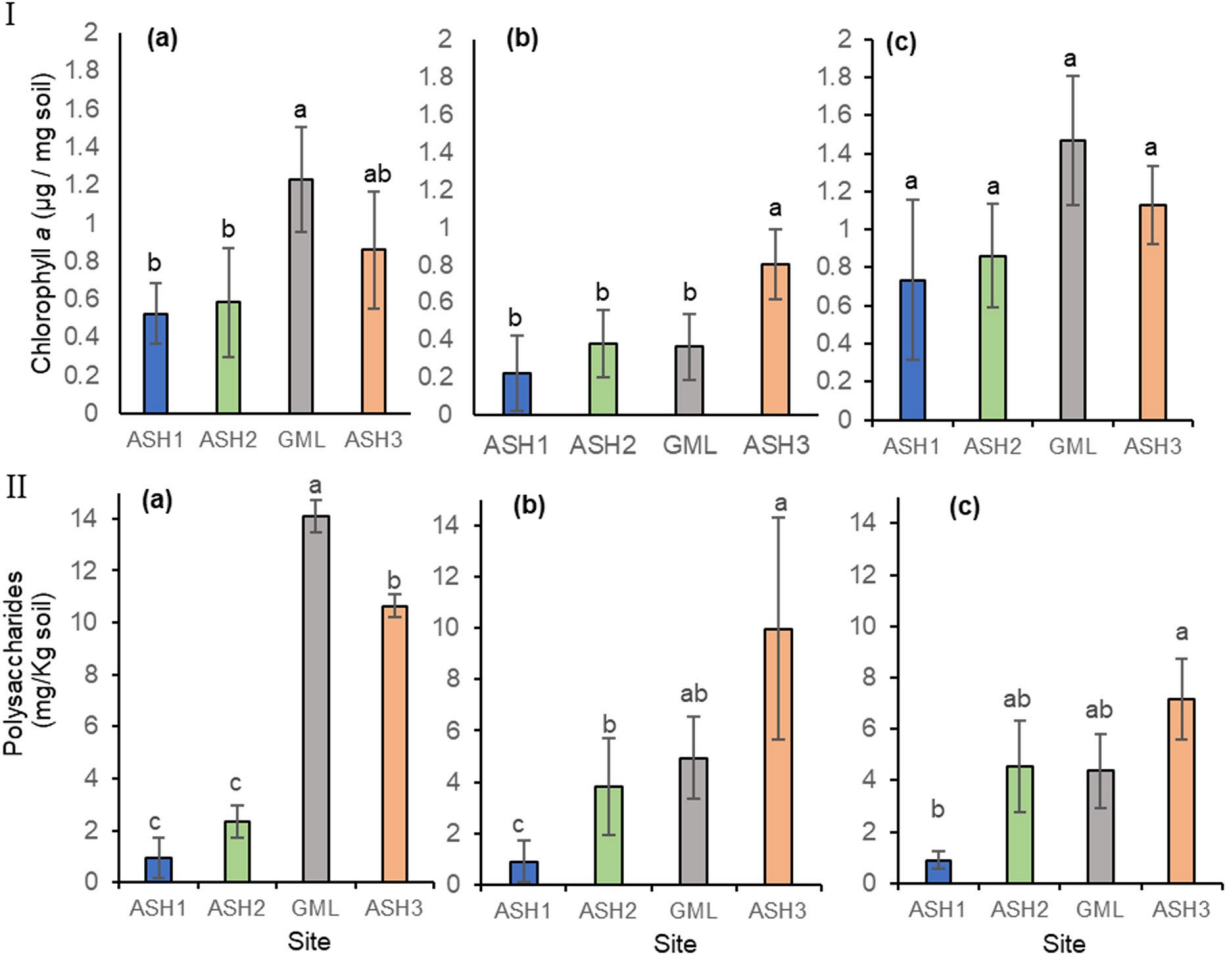
The concentration of chlorophylls (**I**) polysaccharides (**II**) that were extracted from the biocrusts at the four study sites. The reference-sampling date in December 2018 (a) is a reference sample, 2019 (b), and 2020 (c). The columns show the mean ± standard error

The percentage of chlorophyll and polysaccharides concentrations, and land surface coverage (Online Resource Fig. S13) were examined as biocrust indices (Levi et al., 2021; Zaady & Offer 2010, 2016b). The levels of chlorophyll (a) in the soil directly measure the presence or development of photoautotrophic microorganisms in the soil. Any damage to these organisms will be expressed almost directly to their deterioration level (Fig. 7I). Here, we found no statistical differences between sites, although the ASH1 is still smaller than its GML control site, while the ASH2 site showed significant improvement compared to the ASH3 control site. Nevertheless, chlorophyll and polysaccharide levels are improving toward 2020, suggesting possible recolonization (Fig. 7Ic), the ASH1 site still showing low levels over the years (Online Resource Fig. 7II).

Total chlorophyll (*a*) concentrations in ASH1 and ASH2 sites were lower than in GML and ASH3 control sites in 2018 (a) (*p* < 0.001). In 2019 (b), a higher and more significant difference was evident between ASH3 and the other three sites (*p* < 0.001). No differences were obtained in 2020 (c) (Fig. 7I). The ANCOVA test, which was divided according to the type of site, found no significant effect on the four sites’ treatments. However, a significantly lower T1 (*p* < 0.0001) was obtained during the two sampling years (Online Resource Fig. S18).

While chlorophyll and polysaccharide levels are improving toward 2020, suggesting possible recolonization, the ASH1 site still shows low levels over the years (Online Resource Fig. S18 panel II).

Chamizo et al. (2018) reported that cyanobacteria synthesize exopolysaccharides, which increase soil fertility water retention and improve soil structure and stability. The polysaccharides secreted by the cyanobacteria play an important role in the adhesion of the soil particles (Chamizo et al., 2018). The results revealed that although small changes occur (no statistical differences between the ASH1 and GML), continued damage is still evident in the levels of polysaccharides in the ASH1 site. At the same time, polysaccharides in the ASH2 showed an improvement in the recovery phase over time (Fig. 7II).

Similar to the chlorophyll (*a*) concentrations, the concentration of extracted polysaccharides from ASH1 and ASH2 biocrust samples during December 2018 (a) was significantly lower (*p* < 0.0001) than those in the control samples (GML and ASH3). During the following samplings (2019, 2020), the differences were attenuated, and still, the samples of ASH1 (most contaminated) contained the lowest levels (Fig. 7II).

While chlorophyll and polysaccharide levels are improving toward 2020 in the ASH2 site, suggesting possible recolonization (Online Resource Fig. S18), the ASH1 site still shows low levels over the years (Online Resource Fig. S18 panel II b).

A laboratory analysis and field measurements with five (including natural) rehabilitation treatments were used to determine the comprehensive biocrust conditions in the streambed; (1) restoration by application of organic matter in the plot [O], (2) local disruption of the biocrust layer [B], (3) by application of biocrust components collected in the control area [C], (4) natural rehabilitation [N], and (5) rehabilitation by application of the biocrust components isolated and grown in the laboratory [I].

**Fig. 8:**
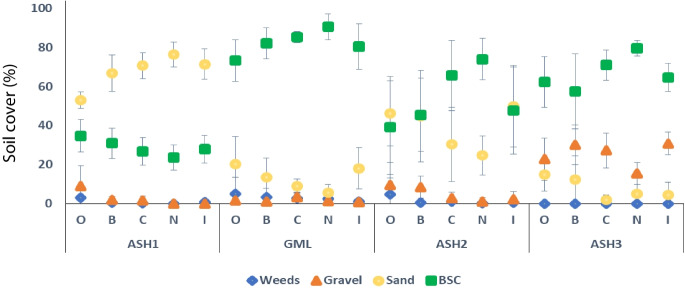
Comparison between biocrust soil coverage at the four research sites as affected by the different treatments 4 years (2020) after the event. Restoration by application of organic matter in the plot (O), local disruption of the biocrust layer (B), by application of biocrust components collected in the control area (C), natural rehabilitation (N), and rehabilitation by application of the biocrust components isolated and grown in the laboratory (I)

The highest biocrust and lowest sand coverages were measured in the control sites. The applied treatments did not affect biocrust coverage in ASH1 (apart from treatment O). Biocrust coverage in the ASH2 alluvial site was comparable to that in the control. It was highest in the N treatment (Fig. 8). It also yielded the highest coverage in both control sites. Within the sandy section, the Oneway Anova test indicated a significant difference (*p* < 0.0001) between ASH1 and GML, with a higher sand level in ASH1 and a lower level of biocrust cover than GML.

The damage to the photoautotrophic organisms, especially at the ASH1 site, raises the concern that there is a limiting factor, whether it is continued physical damage by the migration of the sand or growth-suppressing chemical substances that are still in the soil since the pollution event (Table S1, Table S2). It was reported that a substantial decrease in the biocrust biomass in contaminated soils was observed when the soil was not moist enough during biocrust rehabilitation stages to encourage natural restoration (Wang et al., 2008). Ashalim Nature Reserve is in a hyper-arid region (Fig. S10). Therefore, a critical water shortage resulting from the climate conditions can also be a limiting factor. These issues remain open and require consideration in future research. There is still concern that contamination components (Table S1) may still be present in the soil and affect biocrust development.

To summarize the effect of treatments on acid water-contaminated sites and emphasize which treatments have the potential to re-establish the biocrusts to improve and maintain a rapid process of establishing them, we referred to the result obtained (Fig. 8) regarding the comparison between biocrust soil surface coverage at the four research sites as affected by the different treatments four years (2020) after the event.

While we found a significant improvement at site ASH2 four years after the event, most treatments did not indicate a significant statistical difference compared to the control ASH 3 site. The main problem was found at the ASH1 site.

At the ASH1 site, there were no significant statistical differences between the treatments regarding the percentage of coverage of biocrusts. However, in the treatment [O] in which organic material was added, a significant reduction in the percentage of sandy cover and even a slight increase in the germination of weeds is seen. In the treatments [I] and [C] in the first one, an inoculum of cyanobacteria was added, and in the second [C] added biocrusts collected from the area of the control plot (GML), a minor increase was visible. However, these did not prevent the high percentage of sand coverage. The natural treatment [N], in which no addition was made, showed the lowest result in the coverage percentage of the biocrusts and a high percentage of sandy coverage.

Comparison with the control site (GML) found that in the three parameters, the percentage of ground cover (weeds, sand, and biocrust) was found to be high and statistically significant compared to those of the ASH1 site (Figs. 5Ic, and 8).

Based on our findings, it might be helpful to a combination of treatments [O], [I], and even treatment [C] (in case of a more humid climate) could potentially offer an effective rehabilitation option. This approach warrants immediate and thorough further exploration and could lead to significant improvements in the future.

## Conclusion

The study results show that the biocrust layer covering the ground surface of the stream suffered significant damage, both mechanically due to flow in an abnormal volume and biologically and chemically due to exposure to industrial process effluents. The use of treatments that can help indicated a slight change by enrichment of the organic matter and the cyanobacterial inoculation. Restoring the biocrusts in the hyper-arid region without human intervention may take decades due to the conditions created after the event. Since re-establishing the biocrust layer is an initial step in restoring the ecosystem in the affected area, human intervention can accelerate the rate of biocrust restoration.

### Supplementary information

Below is the link to the electronic supplementary material.

Supplementary file1 (DOCX 2800 KB)
