# Supplementary material for: Monitoring dynamics of biocrust rehabilitation in acid-saturated desert soils
Source: Environ Monit Assess. 2024 Jul 9;196(8):715. doi: 10.1007/s10661-024-12865-y (PMC11233293; doi:10.1007/s10661-024-12865-y)
Supplement: Supplementary file 1 — Supplementary file1 (DOCX 2800 KB) [file 10661_2024_12865_MOESM1_ESM.docx]

**Supplementary information**

**Monitoring dynamics of biocrust rehabilitation in acid-saturated desert soils**

**International Journal of Environmental Science and Technology**

Corresponding author: Zaady E.**, E‐ma**il: [zaadye@volcani.agri.gov.il](mailto:zaadye@volcani.agri.gov.il)

**Appendices**

**Beer-Sheva**

*Egypt*

(Sinai)

200

100

75

50

Eilat

Red

Sea

*Jordan*

300

N

150

*Negev desert*

0 10 20 30 km

*Israel*

Dead

Sea

Med.

Sea

**Ashalim**

**Nature Reserve**

Mediterranean

Sea.

*The Judean desert*

**Figure S9.** The Judean and the Negev Deserts and is an important ecological corridor between these regions. The numbers that appear in the squares indicate isohyets.

**Table S1**. Chemical analysis of the process water in pool No. 3 before the formation of the gap in the wall, during June 2017*.

| Chemical | Symbol | Molecular weight | Average  Concentration  (μ g^-1^) | mg/m^3^ | g/m^3^ |
| --- | --- | --- | --- | --- | --- |
| Boron | B | 10.811 *u* | < 0.1 | < 0.04 | < 0.00 |
| Lead | Pb | 207.2 *u* | 0.13 | 1.1 | 0 |
| Barium | Ba | 137.327 *u* | 0.78 | 4.38 | 0 |
| Arsenic | As | 74.9216 *u* | 2.31 | 7.08 | 0.01 |
| Nickel | Ni | 58.6934 *u* | 2.39 | 5.74 | 0.01 |
| Molybdenum | Mo | 95.94 *u* | 2.89 | 11.34 | 0.01 |
| Cadmium | Cd | 112.411 *u* | 3.23 | 14.85 | 0.01 |
| Titanium | Ti | 47.867 *u* | 3.52 | 6.89 | 0.01 |
| Copper | Cu | 63.546 *u* | 4.15 | 10.72 | 0.01 |
| Chromium | Cr | 51.9961 *u* | 10.44 | 22.2 | 0.02 |
| Vanadium | V | 50.9415 *u* | 14.8 | 30.84 | 0.03 |
| Zinc | Zn | 65.38 *u* | 55.92 | 149.53 | 0.15 |
| Strontium | Sr | 87.62 *u* | 91.91 | 329.37 | 0.33 |
| Chlorine | Cl | 35.453 *u* | 814.42 | 1180.93 | 1.18 |
| Sodium | Na | 22.989769 *u* | 1435.74 | 1349.99 | 1.35 |
| Calcium oxide | CAO | 56.0774 g/mol | 5104.56 | 11707.58 | 11.71 |
| Sulfate | SO_4_ | 96.06 g/mol | 8456.3 | 33223.4 | 33.22 |
| Silicon dioxide | SiO_2_ | 60.08 g/mol | 10092.07 | 24790.58 | 24.79 |
| Iron (III) oxide | Fe_2_O_3_ | 159.69 g/mol | 200 | 1306.26 | 1.31 |
| Aluminum oxide | Al_2_O_3_ | 101.96 g/mol | 300 | 1251.04 | 1.25 |
| Magnesium oxide | MgO | 40.3044 g/mol | 900 | 1483.6 | 1.48 |
| Fluorine | F | 18.998403 u | 18800 | 14608.18 | 14.61 |
| Phosphorus pentoxide | P2O5 | 283.886 g/mol | 18900 | 219445.62 | 219.45 |

*The data cited from a literature report on Nahal Ashalim (Tzohar, 2018). (The letter *u* symbolizes a unified atomic mass).

**Table S2**. The results of the soil nutrient analysis at zero-time (December 2018).

| Saturation (%) | F  (mg/kg) | K (meq/l( | Na (meq/l( | Ca+Mg  (meq/l( | Ca  (meq/l( | P  (mg/kg( | N-NO_2_  (mg/kg) | N-NH_4_  (mg/kg) | PAR (%) | SAR (%) | Site* |
| --- | --- | --- | --- | --- | --- | --- | --- | --- | --- | --- | --- |
| 21.1 | 185.8 | 0.71 | 3.6 | 37.4 | 33.5 | 101 | 0 | 10.1 | 0.16 | 0.82 | ASH1 |
| 18.6 | - | 1.33 | 9.6 | 36.4 | 29.3 | 179.4 | 11 | 6 | 0.31 | 2.24 | ASH2 |
| 17.2 | 5.45 | 4.36 | 16.9 | 33.1 | 23.5 | 16.2 | 5 | 5.5 | 1.49 | 4.09 | GML |

*The alluvial control section was added after the first sampling period**.**


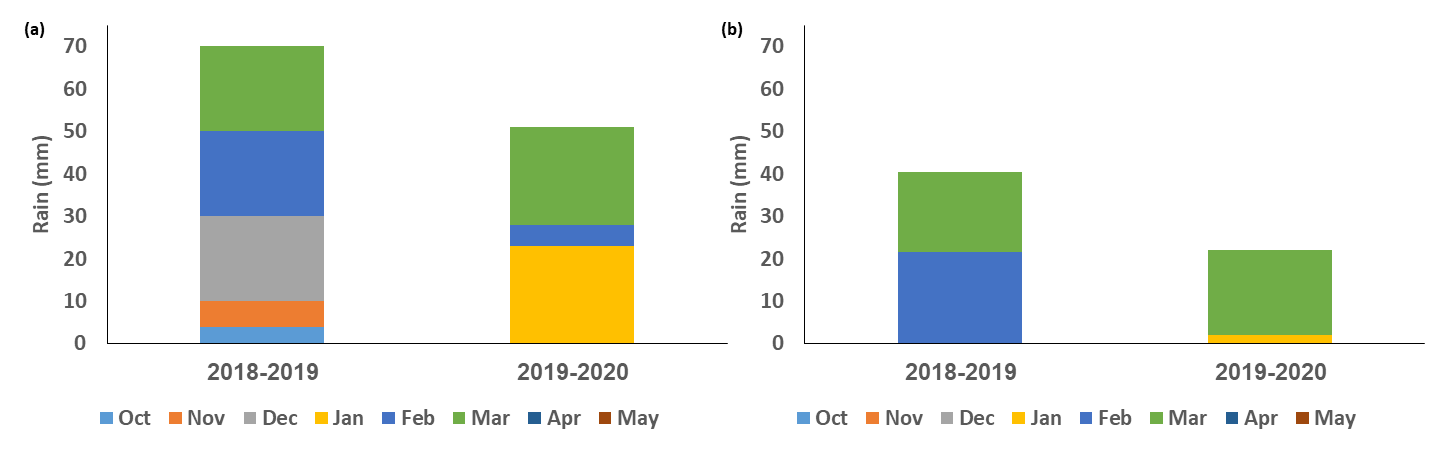


**Figure S10**. Rainfall accumulation during the sampling seasons. (a) Represents the sandy ASH1 and GML sites, and (b) represents the alluvial ASH2 and ASH3 - sites. At both areas, there is a gap of 20 mm of rain between winter 2018-2019 and winter 2019-2020 ([Israeli Meteorological Service](file:///C:\Users\zaadye\Desktop\מאמר%20טל%20אשלים\.%20https:\ims.gov.il\en)).


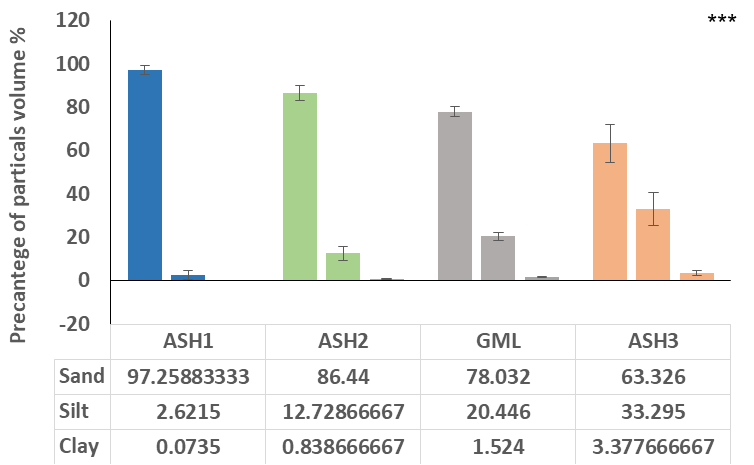


**Figure S11.** The distribution of the soil mechanical composition at the four sites. The columns show mean ± standard error (***p <0.0001).

The mechanical composition consists of three sections, sand, silt and clay. In all the four research sites, there is a significant difference between all sites. The sand component was higher and the clay was very low, even the alluvial section (ASH2 and ASH3) (Online Resource Fig. S3).


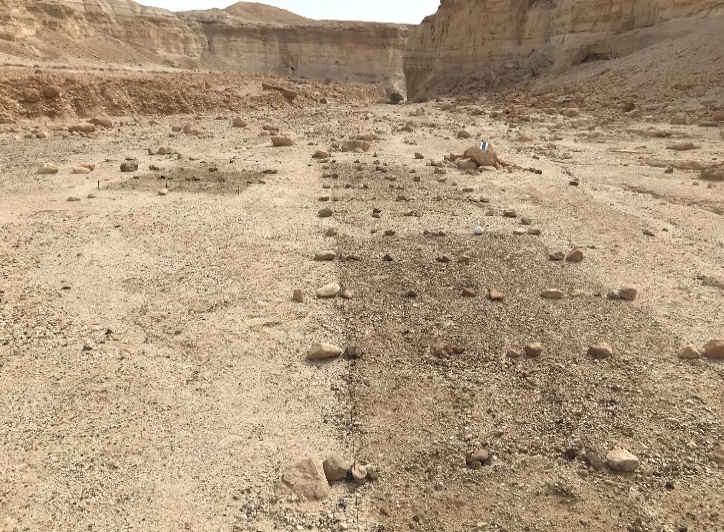


**4**

**3**

**2**

**1**


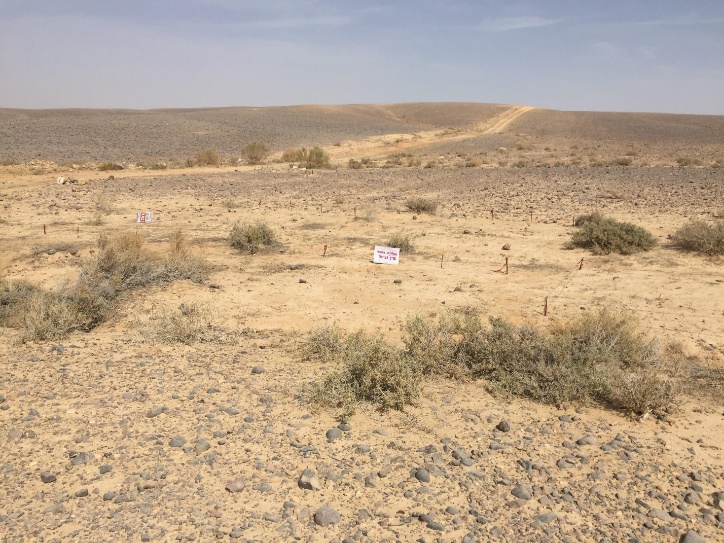

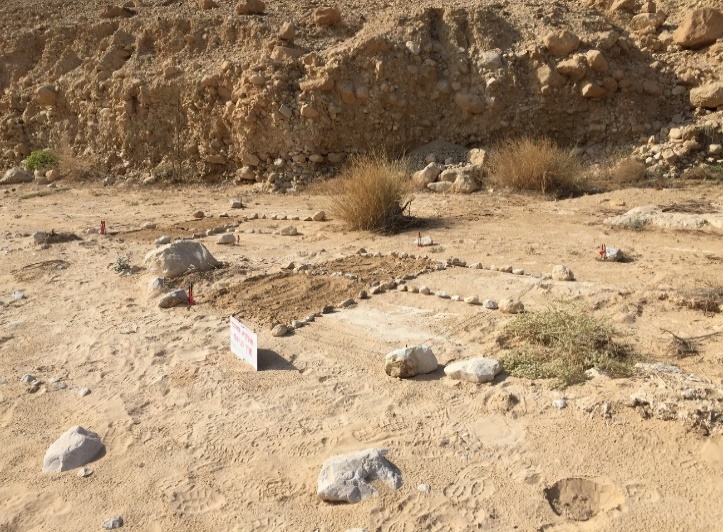

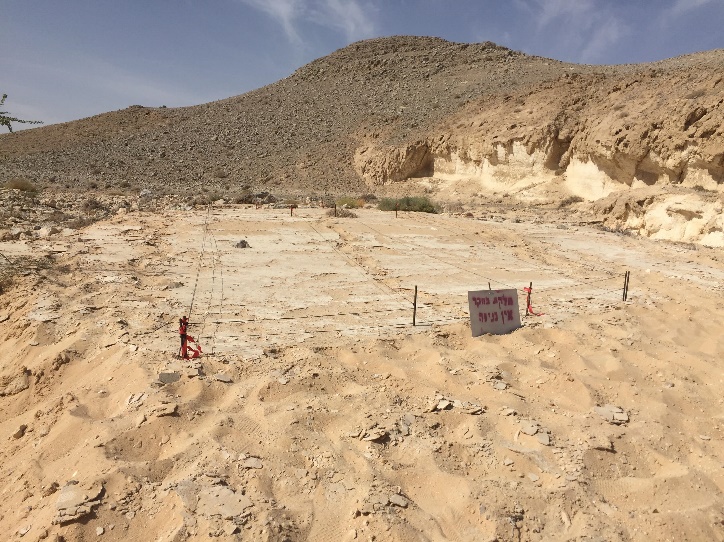


**Figure S12**. Plots at the research sites, 1. The Ashalim sandy ASH1 site, 2. The Ashalim sandy GML site. 3. The Ashalim alluvial ASH2 site, 4. The Ashalim alluvial ASH3 site.


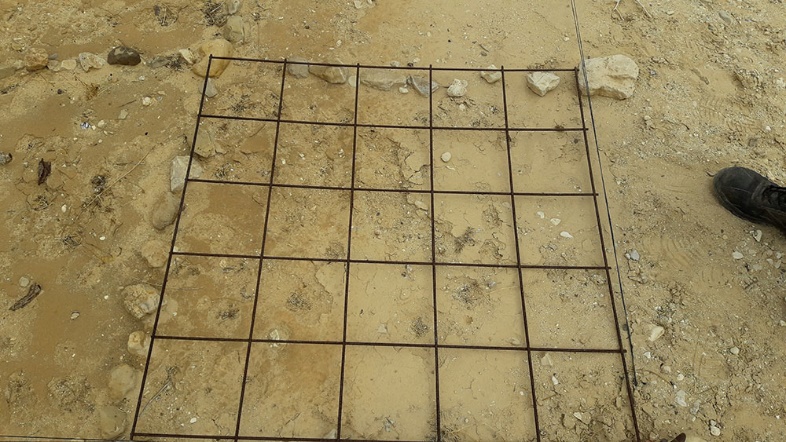


**Figure. S13.** The percentage of coverage test is an observational measure of the biocrusts' presence in the study plots. The test was performed using a metal-mesh, dividing each square meter into 25 equal-sized squares (400 cm^2^). In each 1 m^2^, an assessment was made of the biocrust cover as well as the amount of scale, sand, and vegetation (Belnap et al. 2007).

**I**

**II**

**Figure S14**. The effect of the various treatments on the concentrations of N-NH_4_ (panel I) and N-NO_2_ (panel II). T1 represents the 2019 sampling year results, and T2 represents the results from the 2020 model year. Bars side to side for each site colored the same color as the site but with a different scale of blue, green, grey, or orange: dark scale for treatment [O] followed by [B], [C], [N], and [I] having the lightest scale. The columns show the mean ± standard error.

**I**

**II**

**Figure S15**. Effect of the various treatments on concentrations and the percentage organic carbon (panel I), and potential organic carbon (POC) (panel II). T1 represents the 2019 sampling year results, and T2 represents the results from the 2020 model year. Bars side to side for each site colored the same color as the site but with a different scale of blue, green, grey, or orange: dark scale for treatment [O] followed by [B], [C], [N], and [I] having the lightest scale. The columns show the mean ± standard error.

**Figure S16**. The effect of the different treatments on the electrical conductivity (EC) of the soil surface. T1 represents the 2019 sampling year results, and T2 represents the results from the 2020 model year. Bars side to side for each site colored the same color as the site but with a different scale of blue, green, grey, or orange: dark scale for treatment [O] followed by [B], [C], [N], and [I] having the lightest scale. The columns show the mean ± standard error.

**I**

**II**

**Figure S17**. The various treatments on the shear force (panel I) and penetrability (panel II) of the soil surface. T1 represents the 2019 sampling year results, and T2 represents the results from the 2020 model year. Bars side to side for each site colored the same color as the site but with a different scale of blue, green, grey, or orange: dark scale for treatment [O] followed by [B], [C], [N], and [I] having the lightest scale. The columns show the mean ± standard error.

**I**

**II**

**Figure S18**. Effect of different treatments on chlorophyll (I) (panel I) and polysaccharides (panel II) concentrations. T1 represents the 2019 sampling year results, and T2 represents the results from the 2020 model year. Bars side to side for each site colored the same color as the site but with a different scale of blue, green, grey, or orange: dark scale for treatment [O] followed by [B], [C], [N], and [I] having the lightest scale. The columns show the mean ± standard error.
